# Advanced Magnetic Imprinted Polymers Integrated with In Situ Ionization Mass Spectrometry for High-Throughput Pesticide Screening and Detection in Food Matrices

**DOI:** 10.3390/foods14162786

**Published:** 2025-08-11

**Authors:** Xuan Li, Feng-Lan Lv, Jun-Yun Wang, Yi-Chen Lu, Yun Li, Pan-Pan Li, Min Cao, Ya-Ru Ni, Xiao-Hui Xiong

**Affiliations:** 1 College of Food Science and Light Industry, Nanjing Tech University, Nanjing 211816, China; lyc8844@yeah.net (X.L.); 201961119016@njtech.edu.cn (J.-Y.W.); 202361218248@njtech.edu.cn (Y.L.); 202361218183@njtech.edu.cn (P.-P.L.); xhx@njtech.edu.cn (X.-H.X.); 2 College of Materials Science and Engineering, Nanjing Tech University, Nanjing 211816, China; lvflnjut@163.com (F.-L.L.); nyr@njtech.edu.cn (Y.-R.N.); 3 School of Excellence in Biomedical Engineering, China Pharmaceutical University, Nanjing 211198, China

**Keywords:** magnetic molecularly imprinted polymers, LTP-MS, pretreatment method, multiple pesticide residue simultaneous detection

## Abstract

This research introduces magnetic molecularly imprinted polymers (MMIPs) as a novel tool for the efficient extraction and detection of pesticide residues in food products. The MMIPs exhibit a notable adsorption capacity ranging from 15.70 to 23.57 mg g^−1^, showcasing their efficacy in preconcentrating multiple pesticides. By leveraging Low-Temperature Plasma Mass Spectrometry (LTP-MS) in conjunction with MMIP-based sample pretreatment, the study achieves rapid screening of 108 pesticides in agricultural products, boasting a detection sensitivity of 86.9%. The MMIPs demonstrate exceptional selectivity, enabling swift separation in an external magnetic field, thereby reducing reliance on chemical reagents and facilitating multiple reuses. Rigorous evaluation of the MMIPs’ binding properties, magnetic separation efficiency, and reusability underscores their potential for class-selective enrichment of pesticide residues. The MMIPs were meticulously characterized using a comprehensive array of analytical techniques, including FT-IR spectrometry, SEM, TEM, VSM, and UV–vis spectrophotometry. Remarkably, the MMIPs’ performance in pesticide extraction yielded promising results, with successful qualitative detection of 78 out of 87 identified pesticides in cucumber samples, 71 out of 85 identified pesticides in tomato samples, 55 out of 64 identified pesticides in cabbage samples, and 42 out of 48 identified pesticides in leek samples, achieving recovery rates within the range of 60.12% to 119.84% for 50.91% of the identified pesticides. The screening detection limit (SDL) for the 86 pesticides in the MMMIP-LTP-MS method was set according to the corresponding maximum residue limit (MRL) in the National Food Safety Standard of China (GB 2763-2021). The quantification limits of MMMIPs-LC-TQ-MS ranged from 0.000043 to 5.52 µg g^−1^, with recoveries between 60.12% and 119.84%. These findings underscore the significant impact of MMIP-based sample preparation in enhancing the precision and efficiency of high-throughput determination of pesticide residues in food products.

## 1. Introduction

Pesticides are vital for crop protection and food security in modern agriculture, but their widespread use raises concerns about potential risks to human health and the environment even at relatively low levels. To ensure consumer safety, regulatory bodies across the globe, including those in China, the United States, and the European Union, have implemented maximum residue levels (MRLs) that set strict limits on the permissible concentrations of various pesticide residues allowed on or within food products [1,2,3]. Herein, ever-increasing societal attention and scientific interest have focused on the efficient detection and quantification of trace levels of pesticides in foodstuffs. Subsequently, a diversity of colorimetric, electrochemical, chromatographic, and spectroscopic techniques have been utilized in food analysis [4]. Mass spectrometry (MS) has emerged as an indispensable analytical tool in food analysis, distinguished by its unparalleled sensitivity, specificity, and high-throughput capabilities, which have solidified its position as a cornerstone technique in the field [5]. Among the analytical techniques developed over the past decades, gas chromatography coupled with mass spectrometry (GC-MS) and tandem mass spectrometry (GC-MS/MS) [6], as well as liquid chromatography coupled with mass spectrometry (LC-MS) and tandem mass spectrometry (LC-MS/MS) [7], have emerged as the most widely employed methods due to their exceptional qualitative and quantitative capabilities. The advantages of combining chromatographic separation with mass spectrometric detection are so significant that countries such as China and the United States have adopted GC-MS (or GC-MS/MS) and LC-MS (or L-MS/MS) as the standard techniques for agrochemical analysis and regulation. Despite the advantages of traditional mass spectrometry techniques, their reliance on extensive sample preparation and prolonged analysis times has spurred the development of ambient ionization mass spectrometry (AIMS). This innovative approach enables rapid MS analysis of food products while significantly reducing or even eliminating the need for sample preparation, thereby streamlining the analytical process and increasing sample throughput. While AIMS offers direct analysis capabilities, practical applications often require some sample preparation, especially for complex food matrices. To bridge the gap between the theoretical benefits of AIMS and its practical implementation in complex food matrices, developing an innovative MMIP-AIMS approach seamlessly integrates a streamlined sample preparation process with the direct ionization capability of AIMS, enhancing sample throughput without compromising analytical performance.

Detecting pesticide or hazardous compound residues in foods, particularly fruits and vegetables, is a challenging task due to their complex nature [8]. Pesticide residues often degrade after application or are present in minute quantities, making accurate separation and detection from the intricate food matrix difficult. To overcome this, various pre-treatment techniques are employed for sample clean-up prior to analyzing the target analyte. These techniques include QuEChERS (Quick, Easy, Cheap, Effective, Rugged, Safe), dispersive solid-phase extraction (DSPE), liquid–liquid extraction (LLE), dispersive liquid–liquid microextraction (DLLME), and solid-phase extraction (SPE) [9,10,11,12]. These methods help to isolate and concentrate the desired compounds, facilitating more precise and reliable analysis. However, liquid–liquid extraction (LLE) is a technique that demands the consumption of larger volumes of organic solvents and is considered both time-consuming and labor-intensive. QuEChERS, although widely employed for mycotoxin analysis, is sensitive to matrix types and may lead to significant matrix effects [13]. Solid-phase extraction (SPE) has gained popularity due to its numerous advantages, including a high enrichment factor, efficient removal of interferences, and reduced solvent consumption. In particular, complex food matrices often contain a wide range of interfering compounds, such as lipids, pigments, and proteins, which can hinder accurate quantification of target analytes. SPE’s ability to selectively extract and concentrate target compounds while removing matrix interferences makes it a valuable tool in food analysis. Additionally, SPE’s compatibility with automation and high-throughput workflows is beneficial for routine food safety monitoring and regulatory control. These characteristics make SPE an attractive choice for sample preparation in various analytical applications. However, potential limitations of SPE include the need for careful sorbent selection, potential for sorbent saturation, and the requirement for optimization of extraction conditions. Recently, the molecular imprinting technique has been established as the most effective approach for incorporating targeted molecular recognition sites into the polymer matrix, enabling the creation of highly selective and tailored materials for various applications [14]. Molecularly imprinted polymers (MIPs), a novel SPE material, produced through molecular imprinting technology (MIT), are tailor-made artificial receptors with recognition sites complementary to the template, acting as sorbent materials for target molecules in pesticide detection, and are known for their easy synthesis, high selectivity, and efficient application in harsh environments with high chemo-thermal stability and reutilization across various fields [15].

Magnetic SPE has emerged as a promising alternative to conventional SPE due to several key benefits, including improved extraction efficiency, simplified sample handling, reduced solvent consumption, and increased versatility. Magnetic molecularly imprinted polymers (MMIPs) are a novel and advanced selective extraction technique that builds upon the benefits of MSPE, including enhanced selectivity, improved extraction efficiency, reduced matrix effects, and versatility in synthesis [16]. The selective binding sites in MMIPs provide a high degree of selectivity towards the target analytes, minimizing the co-extraction of interfering compounds from complex food matrices. This leads to stronger interactions between the sorbent and the target analytes, resulting in improved extraction efficiency compared to non-imprinted magnetic sorbents [17]. The selective extraction of target analytes by MMIPs also helps to minimize the co-extraction of matrix components, reducing matrix effects and improving the accuracy of subsequent analytical techniques. Furthermore, MMIPs can be tailored to a wide range of target analytes by selecting appropriate functional monomers and optimizing the imprinting process, resulting in a powerful tool for the selective extraction of target contaminants from complex food matrices.

While various analytical techniques are indeed available, there are still significant challenges and limitations that necessitate the development of more efficient and reliable methods. Our proposed MMIP-AIMS approach addresses these challenges by offering improved selectivity, enhanced sample throughput, and reduced matrix effects, while also being cost-effective and environmentally friendly. By developing MMIPs with high adsorption capacity and reusability, the study seeks to enhance the preconcentration of multiple pesticides from food samples. Furthermore, the investigation aims to demonstrate the utility of Low-Temperature Plasma Mass Spectrometry (LTP-MS) in conjunction with MMIP-based sample pretreatment for rapid screening of a wide range of pesticides in agricultural products. The study also focuses on evaluating the selectivity, magnetic separation efficiency, and reusability of MMIPs, highlighting their potential for class-selective enrichment of pesticide residues. Through a comprehensive characterization of the MMIPs and rigorous analysis of their performance in pesticide extraction, the research aims to provide a novel and effective approach for the detection of pesticide residues in food products. Ultimately, the study aims to contribute to the advancement of rapid, high-throughput, and reliable methods for pesticide residue determination, with a focus on reducing environmental impact and enhancing food safety standards.

## 2. Materials and Methods

### 2.1. Reagents and Apparatus

Dichlorovos (DDV), fluometuron (FMU), and chlorotoluron (CHL) were obtained from Wuhan Yuancheng Gongchuang Technology Co., Ltd. (Wuhan, China). Ferric chloride hexahydrate (FeCl_3_·6H_2_O), ethylene glycol, sodium acetate, and primary secondary amine (PSA) were provided by Sangon Biotech Co., Ltd. (Shanghai, China). Polyethylene glycol (PEG-2000), polyvinyl pyrrolidone (PVP), methacrylic acid (MAA), trimethylolpropane trimethacrylate (TRIM), and 2,2′-azobis (2-methylpropionitrile) (AIBN) were obtained from Shanghai Macklin Biochemical Co., Ltd. (Shanghai, China). Diethylene glycol, 3-hydroxytyramine hydrochloride (DA-HCl), and 3-methacryloyloxypropyltrimethoxysilane (MPS) were acquired from Aladdin Co., Ltd (Shanghai, China). Magnesium sulfate anhydrous (MgSO_4_) and sodium chloride (NaCl) were purchased from Lianyungang Guansu Industrial Co., Ltd. (Lianyungang, China). Tris (hydroxymethyl) aminomethane and octadecylsilane (C_18_) were provided by Shanghai Yansheng Biochemical Reagents Co., Ltd. (Shanghai, China) and Beijing Puhe Biotechnology Co., Ltd. (Beijing, China), respectively. Pesticide standard mix (MRLs in National Food Safety Standard of China, GB 2763-2021) was purchased from Alta Technologies Ltd. (Victoria, Australia) (Table S1) [18]. Methanol (chromatographic-grade), triethylamine, acetonitrile, and ethanol were purchased from Guanghua Technology Co., Ltd. (Haining, China).

Fourier transform infrared (FT-IR) spectrometry (Thermo Fisher Scientific, Waltham, MA, USA) was used for the characterization of the functional groups on the surface of prepared materials. Transmission electron microscopy (TEM, Tecnai 12, Philips, Amsterdam, The Netherlands) and scanning electron microscopy (SEM, S-4800 II, Hitachi, Tokyo, Japan) were employed to observe the morphology and micro-structure, respectively. The magnetic torque of the synthetic nanoparticles was measured using a vibrating sample magnetometer (VSM, Lake Shore 7404, Westerville, OH, USA). The absorption capacity of multi-pesticides in vegetables was measured using UV−vis spectrophotometry (UV-1900, Shimadzu, Kyoto, Japan). The recovery rate was quantified by LC-TQ-MS (Agilent Technologies, Santa Clara, CA, USA). For qualitative analysis, the pesticides were identified using low-temperature plasma mass spectrometry (LTP-MS, TAPI-TOF 1000, Hexin Analytical Instrument, Guangzhou, China).

### 2.2. Preparation of the MMMIPs for Adsorbing Multiple Pesticides

**Figure 1:**
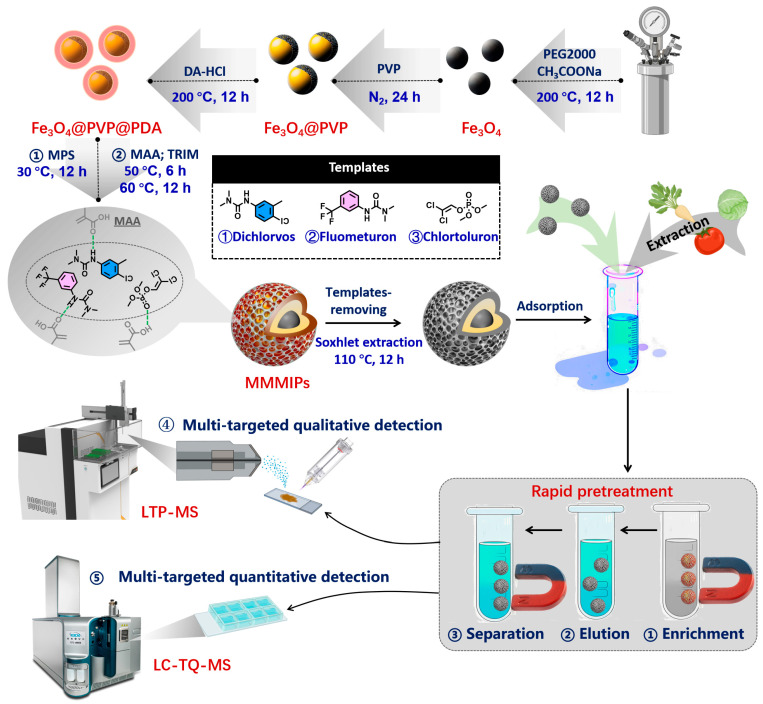
The synthetic pathway for the MMMIPs and the detection process.

The synthesis of MMMIPs involved two primary stages: magnetic core preparation and subsequent surface imprinting polymerization (Figure 1). Using the synthetic method, Fe_3_O_4_ nanoparticles (Fe_3_O_4_ NPs) were obtained according to our previous study with some modification [18,19]. Next, Fe_3_O_4_ NPs were modified by PVP coating (Fe_3_O_4_@PVP). PVP (10 g) was dissolved in distilled water (200 mL) and sonicated for 5 min, followed by the addition of Fe_3_O_4_ (100 mg) and stirring at room temperature for 24 h under N_2_ protection. Then, a solution of dopamine (160 mg) in Tris/HCl (40 mL, 10 mM, pH 8.5) was mixed with a dispersion of Fe_3_O_4_@PVP matrix (70 mg) in 10 mM of Tris/HCl (100 mL) and stirred for 24 h to initiate polymerization under N_2_ protection, forming polydopamine on the surface of Fe_3_O_4_@PVP nanoparticles (Fe_3_O_4_@PVP@PDA). Fe_3_O_4_@PVP@PDA nanoparticles (300 mg) were dispersed in methanol (90 mL), sonicated under nitrogen for 5 min, and then treated with a dropwise addition of MPS solution in dehydrated methanol (25 mL, 1:4 *v/v*) under mechanical stirring at 30 °C for 24 h. The resulting MPS-modified Fe_3_O_4_@PVP@PDA nanoparticles were collected by magnetic separation, vacuum dried at 60 °C for 6 h, and used as magnetic cores for subsequent MMMIP polymerization.

A series of magnetic multi-molecularly imprinted polymers (MMMIPs) were synthesized for the adsorption of various pesticides, using DDV, FMU, and CHL as templates, MAA as a functional monomer, TRIM as a cross-linker, and MPS-modified Fe_3_O_4_@PVP@PDA as a magnetic carrier. Acetonitrile was used as a porogenic solvent in molecular imprinting due to its aprotic and moderately polar nature (ε ≈ 37), which promotes the formation of hydrogen bonds and electrostatic interactions between the functional monomer and template molecules while minimizing interference with the pre-polymerization complex formation, allowing for well-defined recognition sites in the resulting MMIPs. The pre-assembly mixture, containing the magnetic carrier, templates, and functional monomer at various molar ratios (Table S3), was sonicated for 1 h at 30 °C. Subsequently, a solution of TRIM (9 mmol) and AIBN (150 mg) in acetonitrile (30 mL) was rapidly added to the pre-assembly mixture under N_2_ atmosphere, and the polymerization was carried out at 50 °C for 6 h and then at 60 °C for 24 h. The prepared MMMIPs were isolated using an external magnet, and the template molecules were removed by multiple ultrasonic extractions with a methanol-acetic acid solution (2:8, *v/v*). The template molecules were considered to be fully eliminated from the MMMIPs when no detectable levels of the template were found in the supernatant. For comparison, magnetic non-imprinted polymers (MMNIPs) were prepared following the same procedure without the addition of template molecules. The binding capacity (Q_e_) and imprinting factor (IF) were used to optimize the synthetic conditions for the MMMIPs. The equilibrium adsorption capacity (Q_e_, in mg g^−1^) was calculated using Equation (1) [20],

(1) $$Q_{e}=\frac{MV(C_{0}-C_{e})}{m}$$

where *C*_0_ and *C_e_* are the initial and equilibrium template concentrations (mmol L^−1^), *V* is the solution volume (mL), *M* is the template’s molar mass (g mol^−1^), and *m* is the mass of the adsorbent (g). The imprinting factor (IF) was then calculated to evaluate imprinting efficiency using Equation (2) [21],

(2) $$IF=\frac{Q_{MIP}}{Q_{NIP}}$$

where *Q_MIP_* and *Q_NIP_* represent the respective adsorption capacities of the MIP and NIP.

### 2.3. The Adsorption Isotherms of MMMIPs and MMNIPs

To construct the adsorption isotherms, a series of batch experiments were conducted. For each experiment, 10 mg of either the MMMIPs or the MMNIPs was suspended in 5 mL of a methanol-water solution (4:6, *v*/*v*). These solutions contained the target templates—DDV), formetanate (FMU), and chlorpropham (CHL)—at 0.1, 0.2, 0.3, 0.4, 0.5, 0.6, 0.7, 0.8, 0.9, 1.0, 1.25, 1.5, 2.0, 2.5, and 3.0 mmol L^−1^. The resulting suspensions were agitated at 190 rpm and 25 °C for 24 h to ensure that adsorption equilibrium was reached. Following incubation, the magnetic adsorbent particles were isolated from the mixture using an external magnet. The supernatant was then passed through a 0.22 μm syringe filter to remove any remaining particulates. The concentration of the residual templates in the filtrate was subsequently quantified. The concentrations of FMU and CHL were determined by UV–visible spectrometry, measuring absorbance at their respective maxima of 202 nm and 211 nm. Because DDV lacks a suitable UV-absorbing chromophore, its concentration was measured using LC-TQ-MS according to the protocol described in reference [22]. The equilibrium adsorption capacity (*Q_e_*) was calculated from these data using Equation (1), and the results were plotted as adsorption isotherms. To further elucidate the adsorption behavior and surface interaction mechanisms, the experimental data were fitted to the Langmuir and Freundlich isotherm models, presented as Equation (3) and Equation (4), respectively [23].

Langmuir model:

(3) $$\frac{C_{e}}{Q_{e}}=\frac{1}{Q_{m}K_{L}}+\frac{C_{e}}{Q_{m}}$$

Freundlich model:

(4) $$\log Q_{e}=\left(\frac{1}{n}\right)\log C_{e}+\log K_{F}$$

where *C_e_* is the equilibrium concentration of the template (DDV, FMU, or CHL) in the solution (mg L^−1^). *Q_e_* is the equilibrium adsorption capacity of the polymer at concentration Ce (mg g^−1^). *Q_m_* is the theoretical maximum monolayer adsorption capacity of the polymer (mg g^−1^). *K_L_* is the Langmuir constant related to the affinity of the binding sites (L mg^−1^). *K_F_* is the Freundlich constant indicative of the adsorption capacity (mg g^−1^).

### 2.4. Adsorption Kinetics Study of MMMIPs and MMNIPs

To evaluate the adsorption rate and determine the time required to reach binding equilibrium, a kinetic study was performed for both the MMMIPs and the MMNIPs. In a typical experiment, 10 mg of the adsorbent was introduced into 5 mL of template solution (DDV, FMU and CHL) with a fixed initial concentration (1.0 mmol L^−1^). The mixture was continuously agitated at 25 °C. At designated time intervals (1, 2, 3, 5, 10, and 20 min), an aliquot of the suspension was withdrawn, and the magnetic adsorbent was immediately separated from the supernatant using an external magnet. The residual concentration of the template in the supernatant was then measured.

The adsorption capacity at any given time *t*, denoted as Q_t_ (mg g^−1^), was calculated using the following equation:

(5) $${\;Q}_{t}\;=\;\frac{\left(C_{0}\;-\;C_{t}\right)MV}{m}$$

To elucidate the underlying adsorption mechanism and identify the rate-controlling steps, the experimental data were fitted to the pseudo-first-order and pseudo-second-order kinetic models. The linearized forms of these models are presented in Equation (6) and Equation (7), respectively [24].

Pseudo-First-Order Model:

(6) $$\log\left(Q_{e}-Q_{t}\right)=\log Q_{e}-\frac{k_{1}t}{2.303}$$

Pseudo-Second-Order Model:

(7) $$\frac{t}{Q_{t}}=\frac{1}{k_{2}Q_{e}^{2}}+\frac{t}{Q_{e}}$$

where *C*_0_ and *C_t_* (mmol L^−1^) are the template concentrations at the initial time and at the specified intervals (1, 2, 3, 5, 10, and 20 min), respectively. *V* (L) is the volume of the solution, and *m* (g) is the mass of the adsorbent. *Q_e_* and *Q_t_* (mg g^−1^) are the amounts of template adsorbed at equilibrium and at the specified intervals, respectively. *k*_1_ (min^−1^) is the rate constant for the pseudo-first-order model. *k*_2_ (g mg^−1^ min^−1^) is the rate constant for the pseudo-second-order model.

### 2.5. Stability and Reusability Assessment

The operational robustness of the MMMIPs was comprehensively evaluated by assessing both their environmental tolerance and their long-term reusability. To determine their stability under varied environmental conditions, the binding affinity of the MMMIPs was tested across a wide range of pH values (2, 4, 6, 8, and 10) and temperatures (5, 15, 25, 35, and 45 °C), with the equilibrium adsorption capacity (*Q_e_*) calculated for each condition. Furthermore, the recyclability of the polymers was investigated through a series of consecutive adsorption–desorption cycles [25]. In each cycle, 10 mg of MMMIPs was agitated for 10 min in a solution containing 1 mM of a target template (DDV, FMU, or CHL). After magnetic separation, the supernatant was analyzed to determine the adsorption capacity. To prepare the polymers for the subsequent cycle, the bound templates were thoroughly removed via ultrasonic elution with a methanol-acetic acid solution (2:8, *v/v*) until the eluent was free of any detectable template, thereby ensuring complete regeneration before reuse.

### 2.6. The Application of MMMIPs as Pretreatment Materials in High-Throughput Screening of Pesticides in Realistic Agro-Products

Pesticide residues in vegetables, particularly those frequently consumed raw, represent a significant public health concern. To investigate this issue, this study selected four globally popular vegetables—Chinese cabbage, tomato, cucumber, and leek—as representative food matrices [26]. These samples were then screened for non-compliance by evaluating the levels of 108 different pesticides. The screening criteria were the official MRLs stipulated in China’s National Food Safety Standard (GB 2763-2021), with the specific limits for each compound detailed in Table S1. Three sample preparation methods were evaluated for the determination of pesticide residues in the four mentioned vegetable matrices. In all methods, a 4 g portion of each vegetable matrix was weighed into a 15 mL centrifuge tube, followed by the addition of 40 μL of pesticide solution to achieve a concentration equivalent to the MRLs in the sample (Table S1). The samples were allowed to stand for 30 min before proceeding with the respective extraction and cleanup procedures. (1) Detection method without sample pretreatment: Extraction was performed by adding 4 mL of acetonitrile, shaking for 2 min, and sonicating for 5 min. The samples were then centrifuged at 8000 rpm for 5 min, and the supernatant was collected for analysis. (2) Detection method based on QuEChERS pretreatment: Extraction was performed by adding 4 mL of acetonitrile, shaking for 2 min, and sonicating for 5 min. A commercial salt package (0.8 g anhydrous magnesium sulfate, 0.2 g sodium chloride) was added, and the mixture was shaken for 1 min. The samples were then centrifuged at 8000 rpm for 5 min. A 1 mL aliquot of the supernatant was transferred to a commercial cleanup package containing 150 mg anhydrous magnesium sulfate, 25 mg C_18_, and 25 mg PSA. The mixture was then vortexed for 1 min to ensure thorough interaction, followed by centrifugation at 10,000 rpm for 5 min to pellet the sorbents. The supernatant was collected for analysis. (3) Detection method based on MMMIP pretreatment: Extraction was performed by adding 4 mL of methanol and shaking to homogenize the mixture. The samples were centrifuged at 10,000 rpm for 5 min, and the supernatant was transferred to another 15 mL centrifuge tube containing 120 mg of MMMIPs. A 6 mL aliquot of deionized water was added to achieve a methanol-to-water volume ratio of 4:6 in the tube. The samples were shaken at 190 rpm for 30 min at 25 °C. After the reaction, the MMMIPs were magnetically separated, and the supernatant was discarded. The MMMIPs were eluted by sequential sonication with an appropriate volume of acetonitrile. The eluents from the three pretreatments were filtered through a 0.2 µm membrane and prepared for determination by LTP-MS and LC-TQ-MS.

### 2.7. Analysis Conditions of LTP-MS and LC-TQ-MS

To achieve high-throughput rapid screening, samples were first analyzed by LTP-MS (Figure 1), which operated in positive ion generation mode with a helium discharge gas flow rate maintained at 0.8 L min^−1^. Key instrumental parameters were set as follows: the sample loading platform temperature was 180 °C, the capillary temperature was 90 °C, and the capillary voltage was fixed at 130 V. Prior to sample analysis, 100 µg L^−1^ of triazophos was used as a mass calibration compound to ensure high mass accuracy of the instrument. Reliable compound identification criteria were based on the parent ion (*m/z* 314.0728) and characteristic fragment ions (*m/z* 162.0667) of triazophos, requiring a mass error tolerance of less than ±0.05 Da, thereby ensuring the accuracy of analyte identification in high-throughput screening experiments.

For the preliminarily screened samples, LC-TQ-MS was employed for parallel precise quantitative analysis. Chromatographic separation was performed using an ACQUITY UPLC^®^ HSS T_3_ column (100 × 2.1 mm, 1.7 μm) maintained at 40 °C. The mobile phase consisted of a 0.1% formic acid aqueous solution (A) and acetonitrile (B) at a flow rate of 0.4 mL/min, with the following gradient elution program: 0–2 min, 10% B; 2–6 min, 10–90% B; 6–8 min, 90% B; 8–8.1 min, 90–10% B; and 8.1–10 min, 10% B for system re-equilibration. Mass spectrometric analysis was conducted using an electrospray ionization source (ESI) operating in positive ion mode (ESI^+^), with source parameters set as follows: capillary voltage 0.5 kV, desolvation temperature 500 °C, desolvation gas flow 800 L·h^−1^, cone gas flow 150 L·h^−1^, and cone voltage 30 V. Final quantitative analysis was completed using the external standard method.

### 2.8. Statistics

All results are presented as the mean ± standard deviation of triplicate experiments (*n* = 3) and were analyzed for statistical significance by a one-way ANOVA with Tukey’s post hoc test (SPSS Statistics 20), with significance defined as *p* < 0.05.

## 3. Results and Discussion

### 3.1. Preparation of MMMIPs and Their Precursors

The synthetic route for the MMMIPs is illustrated in Figure 1. The interaction between the templates and the functional monomer is a vital factor for the selectivity and adsorbance capability of MIPs. To optimize this interaction, the shift in the UV wavelength of templates after mixing with functional monomers was investigated. As illustrated in Figure S1, the presence of MAA induces a bathochromic shift in the maximum ultraviolet absorption wavelengths of the template molecules FMU and CHL. The magnitude of the red shift is most pronounced when the molar ratio of the template molecule to MAA is 1:6, with a shift of 10 nm and 5 nm observed for FMU and CHL, respectively. This spectral shift suggests the formation of a stable pre-polymerization complex between the template molecules and the functional monomer, MAA, which is a crucial prerequisite for the successful synthesis of molecularly imprinted polymers with high specificity and affinity towards the target analytes. The optimal molar ratio of 1:6 ensures the formation of a sufficient number of non-covalent interactions, such as hydrogen bonding and electrostatic interactions, between the template molecules and MAA, facilitating the creation of well-defined recognition sites within the polymer matrix. The carboxylic acid group of MAA can be considered a hard Lewis base, while many pesticides contain functional groups that can act as hard Lewis acids (e.g., protonated amines) or borderline Lewis acids (e.g., triazines). The hard–hard or hard–borderline acid–base interactions between MAA and the target pesticides contribute to the formation of stable pre-polymerization complexes, leading to improved recognition and binding selectivity in the resulting MMIPs [27]. The molar ratio of templates to MAA equal to 1:6 was selected for MIP preparation, due to its remarkable redshifts. To prepare the dispersive adsorbents, the Fe_3_O_4_ nanoparticles were selected as the core of the MMMIPs. The PVP was used to coat the Fe_3_O_4_ nanoparticles for preventing their agglomeration. PDA, a highly biocompatible monomer, was subsequently wrapped on the surface of PVP-modified Fe_3_O_4_ nanoparticles (Fe_3_O_4_@PDA). To obtain a suitable thickness of PDA layer on the Fe_3_O_4_ nanoparticles, a group of DA-HCl at 67~534 mg was added in the reaction system. As shown in Table S2, the PDA layer became thicker with the increase in DA-HCl. The magnetic property of the prepared Fe_3_O_4_@PDA disappeared with the addition of 534 mg of DA-HCl. Importantly, when 200 mg of DA-HCl was added during the synthesis of Fe_3_O_4_@PDA, the resulting nanoparticles exhibited optimal dispersibility without visible agglomeration. This observation led to the selection of Fe_3_O_4_@PDA, prepared with 200 mg of DA-HCl, as the core carrier for the subsequent molecular imprinting process. MPS as a donor of double bonds was coated on the Fe_3_O_4_@PDA to induce the polymerization reaction. After the addition of the initiator AIBN, the MIPs layer was formed on the surface of MPS-modified Fe_3_O_4_@PDA nanoparticles in the mixture containing template molecule (DDV/FMU/CHL), functional monomer MAA, and cross-linker TRIM at 60 °C under N_2_ protection. In the step of MIP polymerization, the categories of templates, functional monomers, and crosslinkers and their molar ratios were optimized for obtaining better imprinted factors. Obviously, MMMIPs with IF_DDV_ = 1.70, IF_FMU_ = 1.79, and IF_CHL_ = 1.85 were the best; therefore, the scheme in which the proportion of templates, MAA, and TRIM was 1:6:10 was selected for the preparation of MIPs (Table S3).

### 3.2. Characterization of MMMIPs

**Figure 2:**
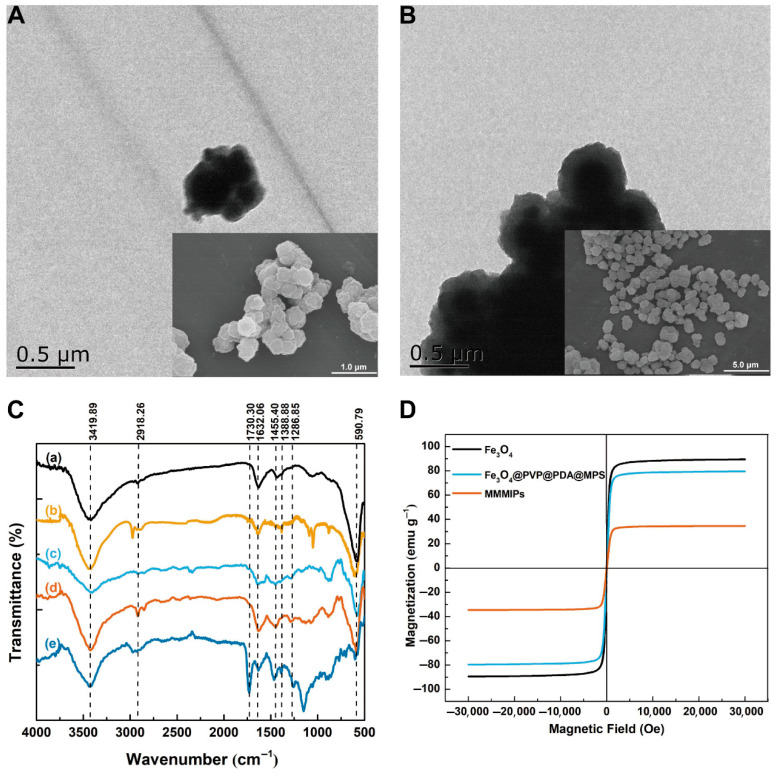
The characterization of the obtained materials. (**A**) The TEM and SEM graph of the MMMIPs. (**B**) The TEM and SEM graph of the MMNIPs. (**C**) FT-IR spectra of Fe_3_O_4_ (line a), Fe_3_O_4_@PVP (line b), Fe_3_O_4_@PVP@PDA (line c), Fe_3_O_4_@PVP@PDA@MPS (line d), MMMIPs (line e). (**D**) Magnetization curves at 25 °C of Fe_3_O_4_, Fe_3_O_4_@ PVP@PDA@MPS, MMMIPs.

The morphologies of the obtained materials were characterized by SEM and TEM. As shown in Figure 2A, Fe_3_O_4_@PVP@PDA nanoparticles exhibit uniform distribution, with an average particle size of 239 nm and PDA coating thickness of around 23 nm. The modification of PDA is able to effectively prevent the oxidation of Fe_3_O_4_ microspheres in air, protect Fe_3_O_4_ from corrosion in acidic solutions, and enhance the biocompatibility [28]. The morphological characteristics of MMMIPs and MMNIPs are illustrated by TEM and SEM in Figure 2A and 2B. The morphology of the MMMIPs exhibited an aggregated, spherical-like structure. Following the modification with PDA and MPS, a light-gray layer was observed surrounding the Fe_3_O_4_ nanoparticles within the MMMIPs (Figure 2A). Furthermore, Figure 2A reveals the growth of a thicker and rougher polymeric shell over the Fe_3_O_4_@PDA@MPS nanoparticles than MMNIPs (Figure 2B), with the imprinted layer encapsulating the spherical particles, resulting in the formation of agglomerated granules. The rough and apparently porous surface of MMMIPs is beneficial in improving their adsorption performance.

To prove the correctness of the synthesis, FT-IR spectra of the obtained materials were registered. In Figure 2C, all materials show a characteristic peak at 590.79 cm^−1^, which was generated by the stretching vibration of Fe-O. This indicated that the imprinted layer was successfully coated onto the Fe_3_O_4_ core. In line (b) of Figure 2C, the evidence of PVP-modified Fe_3_O_4_ nanoparticles is observed at the stretching vibration of C=O at 1632.06 cm^−1^ and the stretching vibration of C-O at 3419.89 cm^−1^. The presence of PDA on the surface of the nanoparticles was confirmed by the characteristic peaks at 1455.40 cm^−1^, corresponding to C-C stretching vibrations, and at 1286.85 cm^−1^, attributed to C-O stretching vibrations and primary amine vibrations in dopamine, as shown in line (c). The successful grafting of MPS onto the ionic surface was evidenced by the C-H stretching vibration at 2918.26 cm^−1^ in line (d). Furthermore, the characteristic peaks at 1388.88 cm^−1^, resulting from the additive methyl symmetric bending vibration, and at 1730.30 cm^−1^, caused by C=O stretching vibration, conclusively demonstrated that the MAA-TRIM layer was successfully polymerized on the surface of the vinyl-grafted magnetic nanoparticles. As shown in Figure 2D, the hysteresis loops of Fe_3_O_4,_ Fe_3_O_4_@PVP@PDA@MPS, and MMMIPs crossed the zero-field line, indicating that all tested materials exhibit superparamagnetic behavior without any residual or coercive magnetization. The saturation magnetization values of Fe_3_O_4_, Fe_3_O_4_@PVP@PDA@MPS, and MMMIPs were found to be 89.5 emu g^−1^, 79.6 emu g^−1^, and 34.6 emu g^−1^, respectively. This gradual decrease in saturation magnetization can be attributed to the increasing presence of non-magnetic material surrounding the magnetic core. Despite this reduction, the MMMIPs in solution could still be rapidly separated under the influence of an external magnetic field, which satisfied the requirements for subsequent experiments. Similar saturation magnetization values have been reported for other magnetic molecularly imprinted polymers, confirming the good magnetic responsiveness of the synthesized MMMIPs [13,17].

### 3.3. Adsorption Behaviors of the MMMIPs and MMNIPs

#### 3.3.1. Static Adsorption

**Figure 3:**
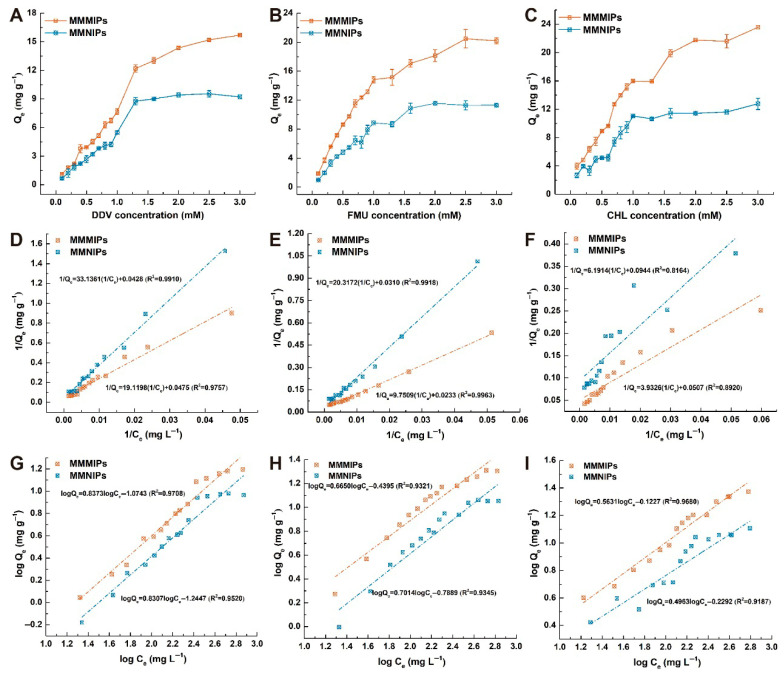
Static adsorption isotherm analysis of DDV, FMU, and CHL on MMMIPs and MMNIPs. Panels (**A**–**C**) show the experimental adsorption isotherm curves for DDV, FMU, and CHL, respectively. The corresponding data were fitted to the Langmuir Isotherm model panels (**D**–**F**) and the Freundlich Isotherm model panels (**G**–**I**).

The binding capacity of MMMIPs to template molecules was investigated using static equilibrium adsorption experiments, which were conducted with initial concentrations of DDV, FMU, and CHL ranging from 0.1 mM to 3.0 mM. As shown in Figure 3A–C, the MMMIPs demonstrated significantly higher pesticide uptake compared to MMNIPs, particularly at higher target pesticide concentrations. At the maximum concentration of 3.0 mM, the calculated imprinting factors (IFs) for DDV, FMU, and CHL were found to be in the range of 1.70–1.85, confirming the superior performance of MIPs over NIPs. To further elucidate the adsorption characteristics of MMMIPs and MMNIPs, the experimental data were fitted using the Langmuir and Freundlich isotherm models. Based on the R^2^ values presented in Figure 3D,E, the adsorption of DDV and FMU on both MIPs and NIPs was better described by the Langmuir model than the Freundlich model. This suggests that the adsorption of DDV and FMU occurs on a limited number of specific localized sites, without lateral interaction or steric hindrance between the adsorbed molecules. In contrast, the adsorption of CHL was slightly better represented by the Freundlich model (Figure 3I), indicating that the adsorption behavior of CHL on the imprinted polymer likely involves multilayer adsorption on a non-uniform surface. Furthermore, the maximum adsorption capacities (Q_m_) of MMMIPs on DDV, FMU, and CHL, as predicted by the Langmuir model, were 21.05, 42.92, and 19.72 mg g^−1^, which were 0.90, 1.33, and 1.86 times greater than those of MMNIPs, respectively (Table S4). It confirmed that imprinted layers on MMMIPs are moderately template-specific. Furthermore, the MMMIPs maintained their adsorption capacity and selectivity in the presence of methanol/water (4:6, *v/v*) solution, allowing the sample preparation process to be greatly simplified. By directly adding water to the methanol extract to achieve the desired methanol/water ratio and then introducing the MMMIPs for adsorption, cleanup, and separation, the need for the rotary evaporation step to remove the organic solvent would be eliminated. This streamlined approach would significantly reduce the overall sample preparation time and improve the throughput of the method, ultimately enhancing the practicality and applicability of the MMIP-based methodology for pesticide determination in food matrices.

#### 3.3.2. Adsorption Kinetics

**Figure 4:**
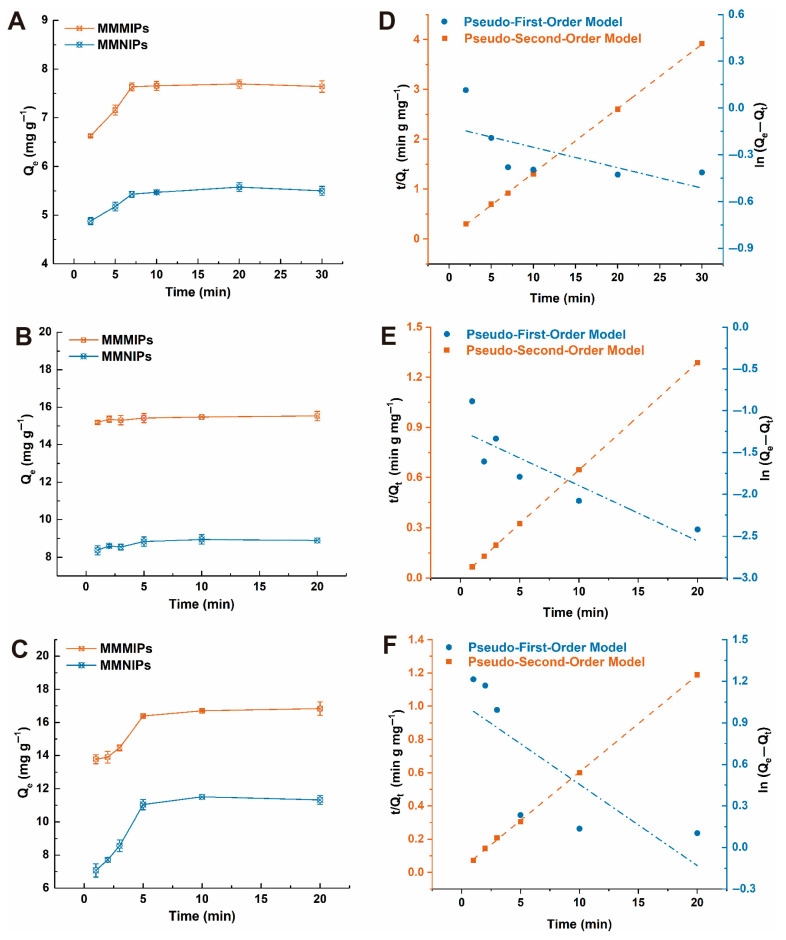
Adsorption kinetics and corresponding model fits for DDV, FMU, and CHL on MMMIPs and MMNIPs. Panels (**A**–**C**) display the time-dependent adsorption profiles for DDV, FMU, and CHL, respectively. The subsequent panels (**D**–**F**) show the fitting of these kinetic data to the pseudo-first-order and pseudo-second-order models for each respective analyte.

To determine the adsorption equilibrium time of the material, the adsorption capacity of MMMIPs towards the mixed template was evaluated at various time intervals using a solution containing DDV, FMU, and CHL, each at a concentration of 1 mM. As depicted in Figure 4A–C, the adsorption equilibrium times for MMMIPs and MMNIPs were investigated at 2.5, 5, 7.5, 10, 20, and 30 min. The results clearly demonstrate that the Qe values of MMMIPs for the three target compounds are significantly higher than those of MMNIPs, which can be attributed to the presence of a greater number of affinity binding sites on the imprinted layer. Interestingly, the adsorption equilibrium times for the three target compounds differ slightly. DDV reaches equilibrium at 7.5 min, while both FMU and CHL achieve equilibrium at 5 min (Figure 4A–C). This variation in equilibrium times can be attributed to the differences in the molecular size, shape, and functionality of the target compounds, which influence their interactions with the imprinted binding sites. The faster equilibrium times observed for FMU and CHL suggest that these molecules have a higher affinity for the imprinted sites and can more readily access and bind to them compared to DDV. The rapid attainment of adsorption equilibrium by MMMIPs is a highly desirable feature for practical applications, as it enables efficient enrichment of the target compounds from food or agro-products extractions in a short time frame. This can be particularly advantageous in water treatment processes, where high throughput and rapid contaminant removal are essential.

To gain deeper insights into the uptake rate of the adsorbate and the potential rate-controlling step, the adsorption kinetics of MMMIPs and MMNIPs were investigated using two well-established kinetic models: the pseudo-first-order equation and the pseudo-second-order equation. The adsorption kinetic constants were determined by fitting the experimental data to these models using nonlinear regression, and the results are summarized in Table S5. The nonlinear regression plots for both models are presented in Figure 4. For both MMMIPs and MMNIPs, the Pseudo-Second-Order Model demonstrates a superior fit compared to the Pseudo-First-Order Model, as evidenced by the higher correlation coefficients (*R*^2^) and the closer agreement between the calculated equilibrium adsorption capacities (*Q_e_*_2,*cal*_) and the experimental values (*Q_e_*_2,*exp*_). This finding strongly suggests that chemisorption is the dominant adsorption mechanism, involving the formation of chemical bonds between the adsorbates and the functional groups on the adsorbent surface. The superior adsorption performance of MMMIPs compared to MMNIPs can be attributed to several mechanistic aspects. Firstly, the presence of specific binding sites created by molecular imprinting enhances the affinity and selectivity of MMMIPs towards the target adsorbates. Secondly, the enhanced surface chemistry of MMMIPs, characterized by a higher density of functional groups, facilitates stronger interactions with the adsorbates. Thirdly, the thin polymer layer and well-defined porous structure of MMMIPs improve mass transfer, enabling faster adsorption kinetics. Furthermore, the spatial arrangement of functional groups in MMMIPs promotes cooperative binding, leading to higher adsorption capacities. Lastly, the selectivity of the imprinted sites in MMMIPs reduces competitive binding, ensuring efficient adsorption even in the presence of interfering compounds [29]. Moreover, the pseudo-second-order rate constants (*k*_2_) of MMMIPs are consistently higher than those of MMNIPs (Table S5), indicating that MMMIPs exhibit faster adsorption rates and larger adsorption capacities. This superior adsorption performance can be ascribed to the synergistic effect of molecular imprinting, enhanced accessibility, multiple interactions, and reduced mass transfer resistance. These factors collectively contribute to the higher adsorption capacities, faster adsorption rates, and improved selectivity of MMMIPs towards DDV, FMU, and CHL compared to MMNIPs, making them a promising adsorbent for the efficient removal of these contaminants from aqueous solutions.

### 3.4. Stability and Regeneration

**Figure 5:**
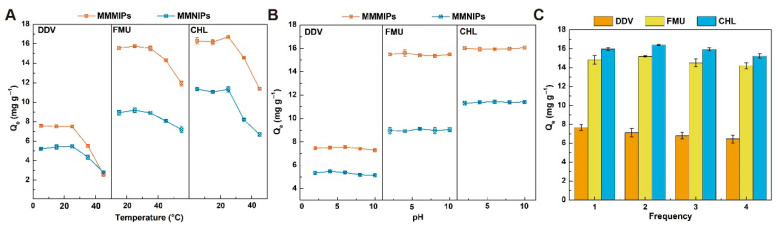
Influence of experimental parameters on the adsorption capacity of MMMIPs and MMNIPs. The performance of polymers under varying conditions of (**A**) temperature and (**B**) solution pH. (**C**) The reusability and stability of the polymers by evaluating their adsorption capacity over multiple consecutive cycles of use.

Environmental tolerance and reproducibility are paramount considerations in the practical application of sorbents [30]. To evaluate the stability of MMMIPs under diverse environmental conditions, their adsorption capacity for the target analyte was investigated in a 1 mM target solution with a pH range of 2–10 and a temperature range of 5–45 °C. As illustrated in Figure 5A, the adsorption of MMMIPs on the target remained relatively consistent from 5 to 25 °C. However, a significant reduction in the adsorption capacity was observed at 35–40 °C. Elevated temperatures can disrupt the non-covalent interactions between MMMIPs and template molecules, leading to the deformation or collapse of recognition sites and reduced specific recognition and selective binding ability, while also increasing non-specific adsorption of target molecules on the polymer matrix, ultimately affecting the sensitivity and selectivity of the extraction [31]. Consequently, 25 °C was identified as the optimal temperature for the application of the material. Figure 5B demonstrates that the adsorption of MMMIPs on the target molecule is unaffected by increasing pH, with both MMMIPs and MMNIPs exhibiting analogous adsorption trends. The adsorption capacity of MMMIPs towards DDV, FMU, and CHL remains stable within the pH range of 2.5 to 10 because these target compounds predominantly exist in their neutral forms and interact with MMMIPs through non-covalent interactions such as hydrogen bonding and hydrophobic interactions, which are less sensitive to the solution pH within the investigated range, considering the pKa values of FMU (4.2) and CHL (4.3) and the absence of ionizable groups in DDV. These findings corroborate the capability of the synthesized imprinted polymers to effectively adapt to both acidic and alkaline environments.

In practical applications, the reusability or reproducibility of solid-phase extraction materials and their cost-effectiveness are additional pivotal factors to consider [32]. Magnetic actuators expedite the regeneration of magnetic MIPs in comparison to conventional MIPs [33]. As depicted in Figure 5C, following four successive adsorption/desorption cycles, the adsorption capacity of MMMIPs can still attain 84–95% of the initial value, and the mass of MMMIPs can be sustained at 91.48% of the original quantity. These observations substantiate that MMMIPs preserve a high adsorption capacity even after multiple uses, with minimal material loss, rendering them suitable for reuse.

### 3.5. Validation and Application of Detection Methods Based on Pre-Processing of MMMIPs in Real Foodstuffs

To ensure food safety amid growing agricultural consumption, rapid and high-throughput screening methods are essential. This approach is designed to reduce the workload and cost of traditional analysis by selectively identifying samples requiring full confirmatory quantification. To support this system, we first constructed an in-house database of 108 pesticides and their corresponding MRLs for four agricultural products, which was then imported into the screening software (Table S1). The screening process involved two key stages. First, for qualitative identification, we established criteria adapted from the SANTE 11312/2021 guideline, where an analyte was considered detected if it met the following conditions: an *m/z* deviation below 0.05 Da (at a mass resolution > 12,000), a fragment ion intensity over 1000, and a signal in the blank matrix below 30%. Second, a sample was flagged as “positive” if a qualitatively identified pesticide exceeded the official MRL as stipulated in China’s national food safety standard (GB 2763-2021).

**Figure 6:**
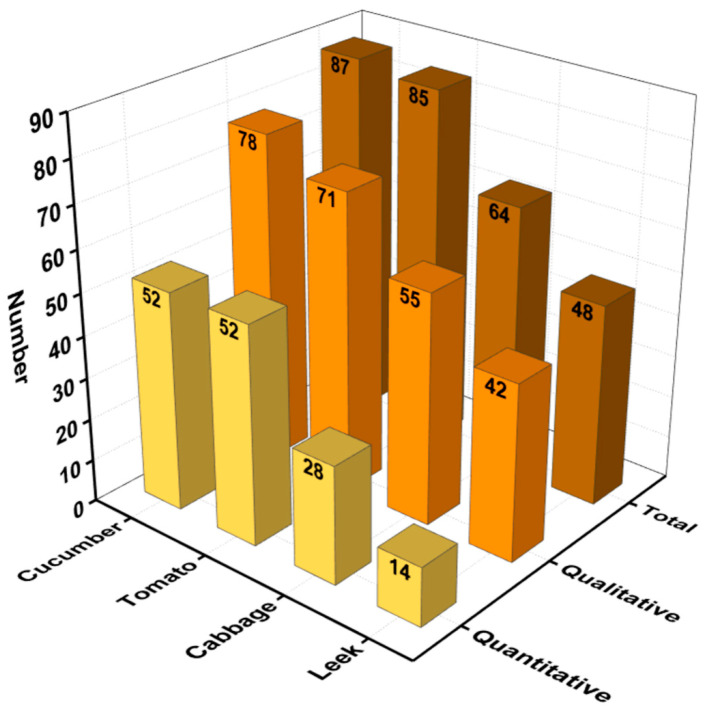
The results of LC-TQ-MS detection with MMMIPs for various pesticides in four vegetable samples.

**Figure 7:**
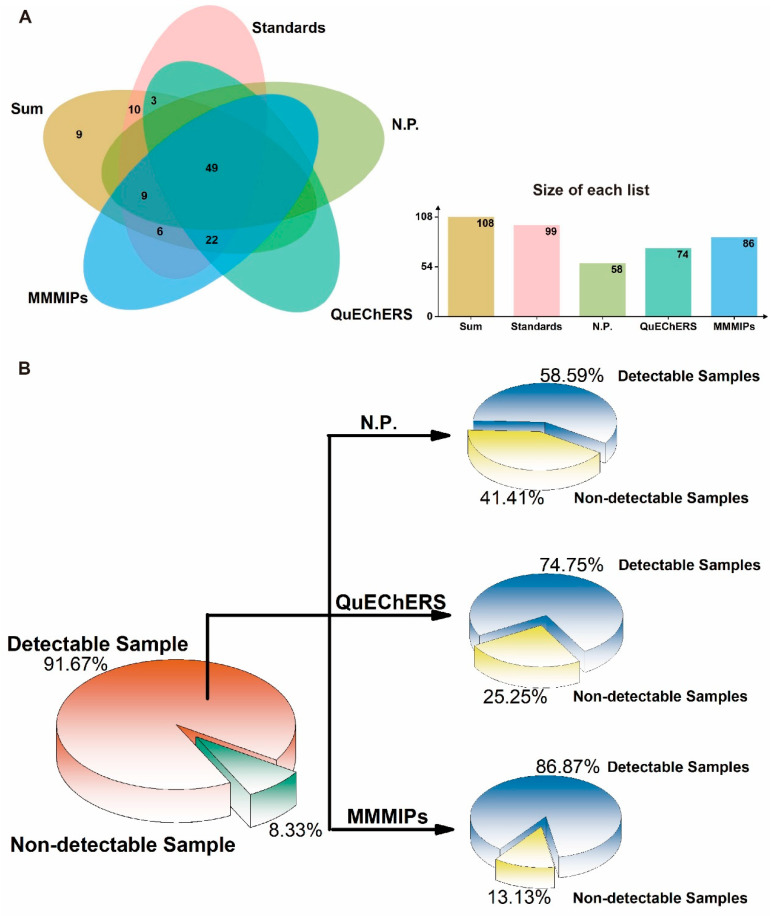
Screening and quantification of pesticides in four agricultural product matrices using different pre-treatment methods. (**A**) The number of pesticides that can be detected simultaneously in four different vegetable matrices using three sample preparation methods. (**B**) Comparison of different pre-treatment methods based on the percentage of pesticides detected from a standard mixture. “sum” represents the total number of pesticides used in the experiment; “standards” indicates the number of pesticide standards that can be detected by the instrument; “N.P.” refers to the sample preparation method that involves only direct extraction without any enrichment or purification steps. The detailed information is shown in Table S1.

The qualitative and quantitative analysis of 108 pesticides in four vegetable samples (cucumber, tomato, cabbage, and leek) was performed using MMMIP-involved sample pretreatment coupled with LTP-MS and LC-TQ-MS. The results, presented in Figure 6 and Table S1, demonstrate the effectiveness of the MMMIP-based method in enhancing pesticide detection compared to non-pretreatment and QuEChERS methods. For the cucumber sample, 78 out of the 87 pesticides required by GB 2763-2021 were detected, with 57 pesticides detected using LTP-MS under MMMIP pretreatment. This represents a 1.97-fold increase in detection compared to the non-pretreatment method. Furthermore, 52 of the 78 pesticides could be quantitatively analyzed, with spiked recoveries ranging from 60% to 130%. Similarly, for the tomato sample, 71 out of 85 pesticides were qualitatively measured using MMMIP-LTP-MS, a 189% increase compared to the non-pretreatment method. Additionally, 52 pesticides were quantitatively determined using MMMIP-LC-TQ-MS. The cabbage sample also showed significant improvement in pesticide detection, with the MMMIP-LTP-MS array detecting 55 out of the 64 required pesticides, a nearly 180% increase compared to non-pretreatment and QuEChERS methods. Moreover, 28 of the 64 pesticides were quantitatively measured using MMMIP-LC-TQ-MS. The leek sample presented a unique challenge due to its low pH, which may affect pesticide adsorption in MMMIPs [34]. Despite this, nearly 88% of the pesticides detected by MMMIP-LTP-MS met the detection requirements of GB 2763-2021, surpassing both non-pretreatment and QuEChERS methods. However, only 29% of the required pesticides could be quantitatively measured using MMMIP-TQ-MS, suggesting that further optimization may be necessary for this specific matrix. Qualitative analysis using LTP-MS revealed that out of the 108 spiked pesticides, 99 pesticide standards could be detected by the instrument. The MMMIP pretreatment method outperformed the others, detecting 86 pesticides (86.87% of the national standard requirements), which was 12 and 28 more than QuEChERS and non-pretreatment methods, respectively (Figure 7). It is particularly noteworthy that the 12 additional pesticides detected by MMMIPs compared to the QuEChERS method were primarily organophosphate pesticides, many of which are highly toxic, such as methidathion and monocrotophos. MMMIP-involved sample pretreatment enabled qualitative determination of nearly 90% and quantitative determination of approximately 60% of the pesticides required by GB 2763-2021 for the studied matrices. These findings highlight the potential of MMMIP-based methods for rapid screening and accurate detection in food safety monitoring.

## 4. Conclusions

A novel method was developed for the efficient, high-throughput monitoring of multiple pesticides in agricultural products, coupling a magneto-actuated SPE with both LTP-MS for screening and LC-TQ-MS for confirmation. Compared to direct extraction (N.P.) and the QuEChERS method, MMMIPs as magnetic absorbents demonstrated rapid isolation and efficient removal of matrix interference, leading to reduced sample preparation time, improved mass spectrometry signals, and minimized signal suppression effects. The MMMIP-based LTP-MS method enabled the qualitative determination of 108 pesticides at default MRLs, as required by the national standards (GB 2763-2021), outperforming other rapid screening methods in terms of the number of pesticides detected, detection time, and applicability to various agro-produce matrices (Table S6). Furthermore, the MMMIP-LC-TQ-MS quantitative detection method allowed for the quantification of 76 pesticides in four different types of vegetables, corresponding to 79% of the requirement-detected pesticides that were qualitatively detected by MMMIP-LTP-MS. The quantification limits of MMMIP-LC-TQ-MS ranged from 0.000043 µg g^−1^ to 5.52 µg g^−1^, with recovery rates between 60.12% and 119.84%, confirming the reliability and robustness of this method (Table S6). Although the MMMIP-LC-TQ-MS quantitative method detects fewer pesticides compared to the QuEChERS-GC/MS/MS approach, it offers distinct advantages in terms of cost-effectiveness and environmental sustainability due to the recyclability of the sample preparation materials. The development of the magneto-actuated SPE coupled with LTP-MS and LC-TQ-MS strategies represents a significant advancement in pesticide monitoring in agricultural products, effectively addressing the challenges associated with matrix interference and enhancing sample preparation efficiency and mass spectrometry performance.

The comparative analysis of the MMMIP-involved LTP-MS method against existing methods highlights its superior performance including detectable amounts, detection time, and applicability to various matrices. These advantages make it an attractive option for fast and in situ admission inspections, addressing the growing need for efficient pesticide monitoring in modern agricultural settings. The quantitative capabilities of the MMMIP-LC-TQ-MS method further demonstrate its potential as a comprehensive tool for pesticide analysis. The ability to quantify a significant portion of the requirement-detected pesticides, along with the wide quantification range and satisfactory recovery rates, underscores the reliability and robustness of this approach. In conclusion, the innovative magneto-actuated SPE coupled with LTP-MS and LC-TQ-MS strategies presented in this study offers a powerful and versatile solution for high-throughput multi-pesticide monitoring in agro-products. The combination of rapid screening capabilities and reliable quantitative analysis makes this approach a promising tool for ensuring food safety and compliance with regulatory standards.

## Data Availability

The original contributions presented in this study are included in the article/Supplementary Material. Further inquiries can be directed to the corresponding authors.

## Supplementary Materials

The following supporting information can be downloaded at: https://www.mdpi.com/article/10.3390/foods14162786/s1. Figure S1: UV spectra of different combinations of DDV, FMU and CHL with different molar ratios of MAA in acetonitrile; Figure S2: The TEM characterization of the obtained materials; Table S1: The number of pesticides qualitatively detected in four types of vegetables using LTP-MS, comparing the sample preparation methods of MMMIPs, QuEChERS, and non-pretreatment (NP); Table S2: Particle thickness of PDA layer; Table S3: The preparative composition of different polymers; Table S4: Adsorption isotherm parameters of DDV, FMU, and CHL onto the MMMIPs and MMNIPs; Table S5: Adsorption kinetics parameters of DDV, FMU and CHL onto the MMMIPs and MMNIPs; Table S6: Performance comparison of MMMIPs as pretreatment materials for pesticide detection with other similar studies. References [35,36,37,38,39,40] are cited in the supplementary materials.
