# Supplementary material for: Advanced Magnetic Imprinted Polymers Integrated with In Situ Ionization Mass Spectrometry for High-Throughput Pesticide Screening and Detection in Food Matrices
Source: Foods. 2025 Aug 11;14(16):2786. doi: 10.3390/foods14162786 (PMC12385875; doi:10.3390/foods14162786)
Supplement: Supplementary file 1 [file foods-14-02786-s001.zip › foods-3752980-Supplemental Materials.pdf]

Supplementary Materials

Advanced Magnetic Imprinted Polymers Integrated with In-situ Ionization Mass Spectrometry for High-Throughput Pesticide Screening and Detection in Food Matrices

Xuan Li [§, 1]; Feng Lan Lv [ξ, 1]; Jun Yun Wang[§]; Yi Chen Lu [§, Ψ, *]; Yun Li [§]; Pan Pan Li [§]; Min Cao [§, *]; Ya Ru Ni [ξ]; Xiao Hui Xiong [§]

[§] College of Food Science and Light Industry; Nanjing Tech University, Nanjing 211816, China

[Ψ] School of Excellence in Biomedical Engineering, China Pharmaceutical University, Nanjing 211198, China

[ξ] College of Materials Science and Engineering; Nanjing Tech University, Nanjing 211816, China

[1] The authors made an equal contribution to the study

[*] Corresponding authors at Puzhu south street No.30, College of Food Science and Light Industry, Nanjing Tech University, Nanjing 211816, China

E-mail address: yichenlu@njtech.edu.cn (Y. C. Lu); anniecao2001@163.com (M. Cao)

TABLE OF CONTENTS

Figure S1. ....Error! Bookmark not defined.

Figure S2. ....Error! Bookmark not defined.

Table S1. ....Error! Bookmark not defined.

Table S2. 17

Table S3. 18

Table S4. 19

Table S5. 20

Table S6. 21

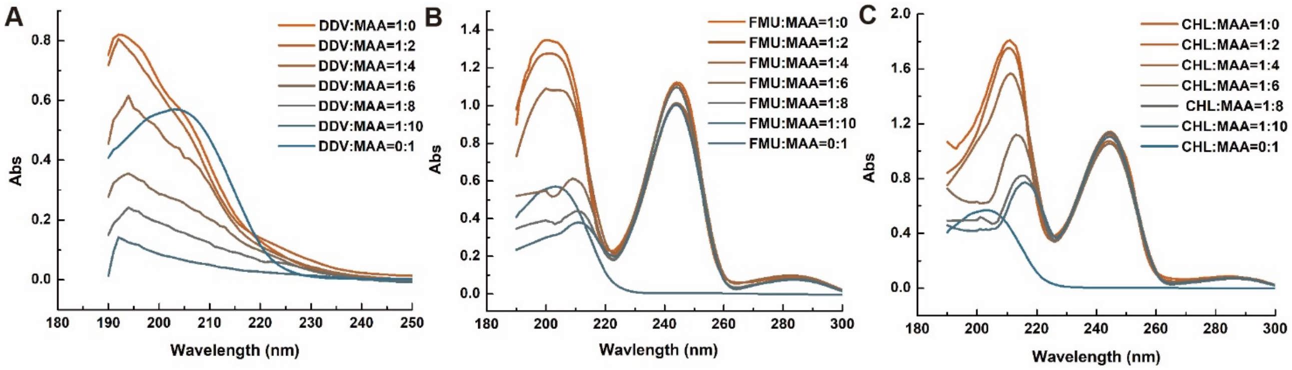

Figure S1. UV spectra of different combinations of DDV, FMU and CHL with different molar ratios of MAA in acetonitrile, respectively. MAA concentration equal to 0.6 mmol L-1; Concentration of pesticides added were 0.3, 0.15, 0.075, 0.05, 0.0375, and 0.03 mmol L-1, respectively. Corresponding pure MAA solutions as blanks or background, which are deducted in each experimental group.

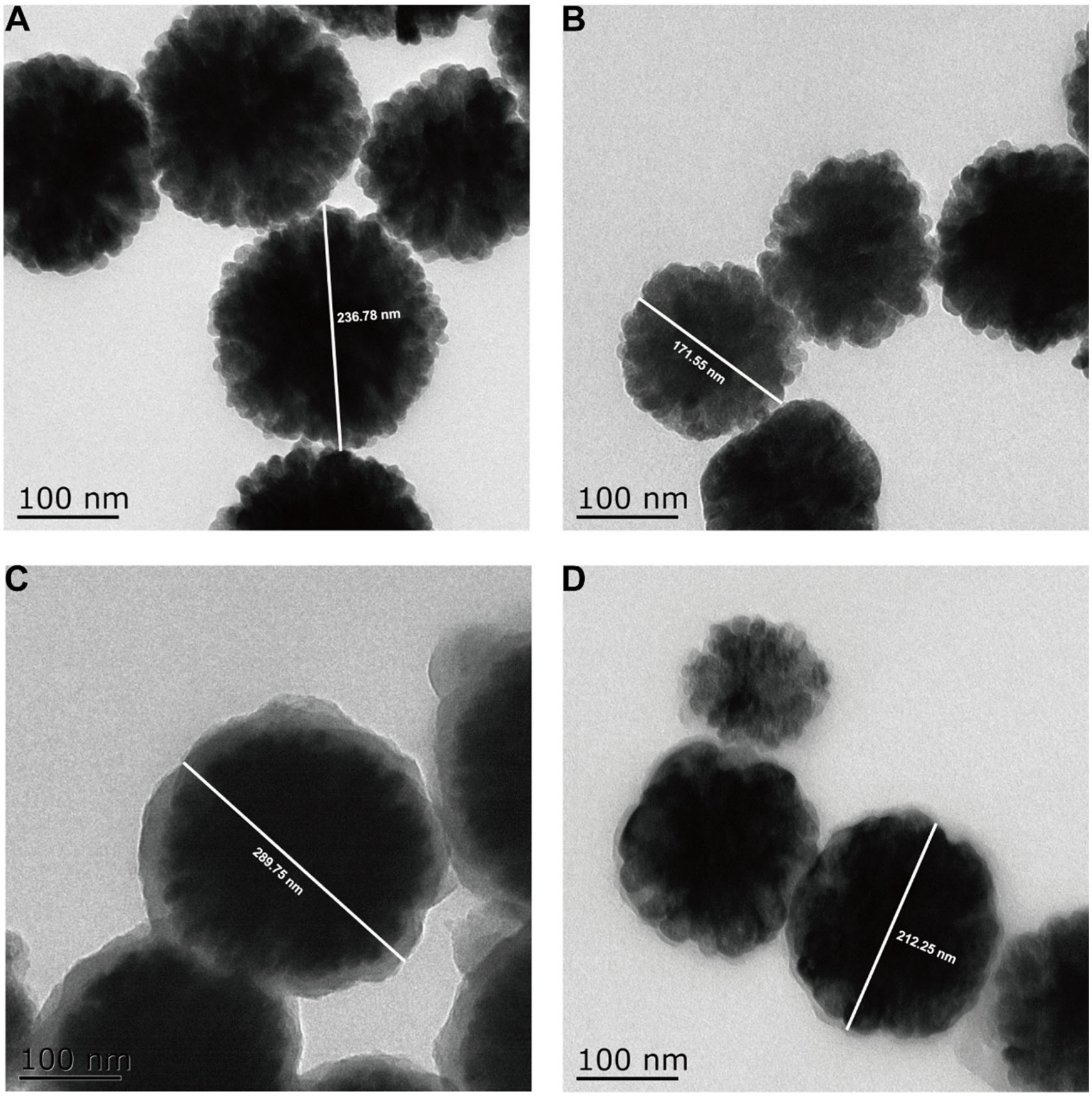

Figure S2. The TEM characterization of the obtained materials, Fe_3O_4 (A), Fe_3O_4@PVP (B), Fe_3O_4@PVP@PDA (C), and Fe_3O_4@PVP@PDA@MPS (D).

Table S1. The number of pesticides qualitatively detected in four types of vegetables using LTP-MS, comparing the sample preparation methods of MMMIPs, QuEChERS, and non-pretreatment (NP). The recovery rates of pesticides quantitatively determined by LC-TQ-MS in four types of vegetables are also presented, comparing the sample preparation methods of MMMIPs and non-pretreatment (NP).

Samples	Chemicals ID	MRLs[a] (mg kg^{-1})	Spiked content (mg kg^{-1})	LTP-MS			LC-TQ-MS				
				N.P.	QuEChERS	MMMIPs	DLs (mg kg-1)	MMMIPs Recovery (%)	RSD	N.P. Recovery (%)	RSD
cucumber	demeton (o+s)	0.02	0.02					41.35	9.29	5.43	54.67
	methomyl	0.2	0.2		√	√	5.03E-02	26.79	2.10	20.48	20.50
	methamidophos	0.05	0.05					2.30	12.97	0.71	4.39
	methidathion	0.05	0.05			√		57.29	23.50	3.96	41.24
	chlordimeform	0.01	0.01	√	√	√	3.22E-02	173.41	17.65	257.02	22.81
	omethoate	0.02	0.02		√		4.03E-03	8.07	20.20	6.71	173.21
	carbofuran	0.02	0.02			√	1.82E-03	63.68	1.91	6.65	33.81
	monocrotophos	0.03	0.03			√	1.16E-01	82.91	15.41	364.33	6.06
	phosfolan-methyl	0.03	0.03		√	√	1.28E-02	20.15	15.03	36.47	15.65
	isofenphos-methyl	0.01	0.01		√	√		148.44	56.05	0	#DIV/!
	ethoprophos	0.02	0.02		√		3.90E-03	112.35	7.75	13.88	37.66
	phosfolan	0.03	0.03		√	√	5.97E-03	30.60	7.58	16.06	21.37
	phorate	0.01	0.01					56.19	2.98	2.47	70.51

	cadusafos	0.02	0.02			√	1.86E-03	100.91	2.76	6.09	47.78
	fenthion	0.05	0.05				1.53E-03	42.55	3.71	1.82	60.01
	parathion	0.01	0.01					55.29	15.33	1.05	131.81
	isazofos	0.01	0.01			√	6.05E-04	98.33	1.48	4.05	45.10
	sulfotep	0.01	0.01				4.31E-04	103.82	1.56	2.88	47.79
cucumber	fipronil	0.02	0.02					148.44	56.05	0	#DIV/!
	aldicarb	0.03	0.03				2.70E-03	38.65	3.49	9.44	48.21
	oxamyl	0.1	2		√		8.66E-01	50.85	11.08	44.59	12.57
	propamocarb	0.3	5	√		√	5.04E-01	27.24	0.65	10.00	14.06
	isoprocarb	0.5	0.5				4.22E-02	84.71	1.23	9.36	37.37
	carbaryl	1	1	√	√	√	1.40E-01	82.76	11.38	14.89	27.42
	permethrin	1	0.5		√	√					
	cypermethrin	1	0.2								
	thiophanate-methyl	0.1	2		√	√	2.02E-01	38.31	2.53	10.10	21.01
	pyraclostrobin	0.02	0.5	√	√	√	4.04E-02	84.25	1.27	8.80	43.41
	dinotefuran	2	2		√	√	5.47E-01	25.20	3.16	28.55	11.74
	imidacloprid	0.2	1		√	√	1.13E-01	69.30	2.97	12.25	25.14
	dichlorvos	0.2	0.2				2.22E-02	49.11	4.77	11.27	22.62
	carbosulfan	0.05	0.2	√		√	1.89E-03	84.14	6.71	1.67	93.70
	acetamiprid	0.2	1	√	√	√	1.32E-01	59.16	2.09	14.06	24.05
	cyhalothrin	0.2	1								
	diethofencarb	1	5	√	√	√	6.28E-01	80.34	2.32	12.88	30.51
	pirimicarb	0.01	1	√	√	√	1.60E-01	64.73	1.08	16.38	24.51
	forchlorfenuron	0.05	0.1		√	√	1.10E-02	92.87	5.42	11.82	29.61

	thiacloprid	0.02	1		√	√	9.53E-02	67.89	3.18	9.90	24.94
	trichlorfon	0.2	0.2				5.23E-02	27.93	16.87	25.98	19.89
	fenitrothion	0.2	0.5				5.00E-02	68.27	23.71	11.28	50.04
	metalaxyl	0.05	0.5	√	√	√	9.31E-02	78.15	1.34	18.99	23.72
	thiamethoxam	0.2	0.5		√		6.37E-01	73.85	6.91	137.49	21.49
cucumber	amitraz	0.5	0.5		√		9.60E-03	25.22	5.07	2.58	72.39
	bifenazate	0.5	0.5		√	√	1.14E-02	30.50	5.05	3.11	49.35
	kresoxim-methyl	0.5	0.5			√	2.04E-02	93.92	4.77	4.81	44.75
	zoxamide	0.02	2	√	√	√	1.01E-01	75.70	4.62	5.64	43.55
	boscalid	2	5	√	√	√	5.00E-01	85.54	1.23	10.33	36.67
	enestroburin	1	1	√	√	√	6.62E-02	100.74	0.66	7.23	50.51
	azoxystrobin	0.1	0.5	√	√	√	6.56E-02	98.07	3.84	13.63	29.49
	trifloxystrobin	0.1	0.3		√	√	1.09E-02	90.54	1.60	4.02	52.73
	bromopropylate	0.5	0.5								
	fenvalerate	3	0.2								
	phoxim	0.05	0.05	√		√	1.59E-03	67.45	8.82	3.29	44.35
	cyromazine	0.5	1	√	√	√	7.18E-02	28.61	9.12	6.83	8.10
	acephate	0.3	1	√	√		2.09E-02	4.29	16.46	2.01	13.10
	chlorpyrifos	0.05	0.1	√	√	√	2.80E-02				
	pyrimethanil	0.05	2				3.21E-01	119.84	7.72	13.89	89.40
	pymetrozine	0.02	1	√	√	√	1.14E-01	80.64	7.45	16.48	25.27
	cyprodinil	0.2	0.2				1.78E-02				
	isocarbophos	0.05	0.05	√	√	√		31.68	9.78	11.06	3.29
	procymidone	0.2	2	√	√		9.36E-02	79.40	6.94	9.28	34.85
	penconazole	0.1	0.1				8.02E-03				

	myclobutanil	1	1		√	√	9.53E-02	70.24	5.49	4.71	17.91
	triadimefon	0.05	0.1		√	√	7.67E-03	96.30	11.25	8.42	37.46
	triadimenol	0.2	0.2		√	√	2.48E-02	96.26	8.61	9.88	33.75
	imazalil	0.5	0.5		√	√	4.73E-02	98.15	11.89	6.85	32.94
	clofentezine	0.5	0.5	√	√	√	2.08E-02	92.25	15.37	12.54	29.00
cucumber	diazinon	0.01	0.1	√	√	√	5.86E-03	65.52	7.43	9.87	27.71
	buprofezin	0.5	0.7			√	6.10E-02	63.98	13.73	4.35	43.13
	tebuconazole	0.05	1	√	√	√	9.97E-02	97.27	9.47	6.11	39.52
	hexaconazole	0.1	1		√	√	9.74E-02	106.90	14.62	8.65	62.64
	flusilazole	0.01	10	√	√	√	7.14E-02	97.83	8.96	9.96	31.67
	malathion	0.2	0.2	√	√	√	1.13E-02	96.17	10.51	10.13	33.19
	fenbuconazole	0.05	0.2	√	√	√	1.91E-02	98.92	8.69	7.24	38.68
	bitertanol	0.5	0.5				5.74E-02	99.74	6.97	6.03	42.76
	hexythiazox	0.05	0.05		√	√	4.50E-03	98.40	9.13	6.62	41.03
	etoxazole	0.5	0.02		√	√	1.81E-03	79.73	8.18	7.15	53.08
	pyridaben	0.1	0.1				3.93E-03	123.43	2.53	5.23	63.11
	prochloraz	0.1	1		√	√	1.54E-01	119.26	12.82	5.99	47.09
	dimethomorph	0.05	5		√	√	1.14E+00	74.82	6.21	2.70	47.35
	difenoconazole	0.02	1		√	√	8.43E-02	92.01	9.15	11.61	31.52
	chlorfenapyr	10	0.5	√	√	√		92.58	7.67	18.52	21.88
	spirodiclofen	0.5	0.07	√	√	√	1.62E-03	86.08	10.78	5.99	38.19
	fenpyroximate	0.1	0.3			√	2.72E-02				
	parathion-methyl	0.02	0.02				1.78E-02	110.69	4.90	0.98	121.57
	carbendazim	0.02	1	√	√	√		127.27	23.27	5.78	50.94

	tolylfluanid	1	5					118.07	18.61	118.60	22.04
Tomato	demeton (o+s)	0.02	0.02					36.95	20.35	7.62	62.51
	methomyl	0.2	0.2		√	√	4.07E-02	26.06	3.17	18.84	8.01
	methamidophos	0.05	0.05					2.36	27.82	4.27	14.46
	methidathion	0.05	0.05			√		122.35	5.54	5.47	11.18
Tomato	chlordimeform	0.01	0.01	√	√	√	2.89E-02	216.00	3.24	283.24	3.99
	omethoate	0.02	0.02					6.57	9.74	13.71	173.21
	carbofuran	0.02	0.02				2.66E-03	85.77	4.79	11.30	16.67
	monocrotophos	0.03	0.03			√	6.45E-02	41.84	12.51	210.15	7.39
	phosfolan-methyl	0.03	0.03		√		1.29E-02	27.83	2.21	41.70	8.25
	isofenphos-methyl	0.01	0.01			√					
	ethoprophos	0.02	0.02		√		3.14E-03	94.67	2.74	13.64	13.31
	phosfolan	0.03	0.03		√	√	5.81E-03	31.29	2.51	18.31	7.38
	phorate	0.01	0.01					84.30	4.17	2.65	30.14
	cadusafos	0.02	0.02				2.13E-03	95.46	1.28	8.51	21.81
	fenthion	0.05	0.05				1.55E-03	79.65	7.35	2.56	18.37
	parathion	0.01	0.01					86.24	35.02	14.09	81.93
	isazofos	0.01	0.01			√	6.41E-04	96.71	5.93	3.46	24.67
	sulfotep	0.01	0.01				4.41E-04	106.53	3.26	5.32	18.28
	propamocarb	0.3	2	√		√	3.33E-01				
	fipronil	0.02	0.02					5.84	37.48	0.53	121.86
	aldicarb	0.03	0.03								
	oxamyl	0.1	2		√			19.88	17.70	15.81	8.90

	fenpropathrin	0.2	1			√	4.28E-02				
	clothianidin	0.5	1		√	√	7.03E-02	81.79	15.85	7.97	10.67
	tebufenozide	0.5	1	√	√	√	1.23E-01	90.28	7.96	11.60	21.36
	carbaryl	1	1	√	√	√	1.80E-01	80.65	9.73	15.04	23.04
	picoxystrobin	0.05	1	√	√	√	9.38E-02	81.66	15.09	7.43	23.97
Tomato	permethrin	1	1	√	√	√					
	cypermethrin	1	0.5								
	cyfluthrin	0.01	0.2								
	thiophanate-methyl	0.1	3	√	√	√	4.82E-01	46.40	2.06	14.27	16.20
	pyraclostrobin	0.02	1		√	√	1.45E-01	105.33	8.28	11.95	23.53
	imidacloprid	0.2	1		√	√	1.51E-01	65.33	9.68	13.61	14.23
	carbosulfan	0.05	0.1				9.57E-04	56.17	21.21	0.50	85.02
	acetamiprid	0.2	1		√	√	1.47E-01	53.24	4.29	13.27	15.89
	cyhalothrin	0.2	0.5								
	diethofencarb	1	1	√	√	√	1.50E-01	104.78	5.16	12.25	24.11
	pirimicarb	0.01	0.5	√	√	√	7.25E-02	43.15	87.81	16.79	11.95
	thiacloprid	0.02	0.5		√	√	4.52E-02	55.76	4.21	8.01	18.93
	deltamethrin	0.5	0.2								
	metalaxyl	0.05	0.5		√	√	1.11E-01	76.92	4.39	19.75	14.99
	thiamethoxam	0.2	1		√		3.63E-01	37.69	115.76	34.20	7.92
	amitraz	0.5	0.5				6.23E-03	10.58	10.01	0.77	59.14
	bifenazate	0.5	0.5		√	√	6.61E-02	96.25	4.39	10.89	22.54
	kresoxim-methyl	0.5	1				1.16E-01	107.21	3.79	9.42	27.57
	zoxamide	0.02	2	√	√	√	1.98E-01	105.37	8.88	7.78	27.46

	boscalid	2	2	√	√	√	2.76E-01	96.73	1.84	11.10	24.60
	azoxystrobin	0.1	3		√	√	5.87E-01	105.26	1.69	16.74	18.72
	trifloxystrobin	0.1	0.7		√	√	5.72E-02	106.26	2.36	6.03	39.45
	fenvalerate	3	0.2					82.90	22.96	3.17	32.63
	flucythrinate	0.05	0.2								
Tomato	novaluron	0.01	0.02								
	phoxim	0.05	0.05		√		3.18E-03	74.49	23.63	5.05	23.82
	acephate	0.3	1	√	√	√	1.24E-02	1.54	9.29	1.36	17.40
	chlorpyrifos	0.05	0.5				2.47E-02	88.57	16.53	3.60	35.03
	pyrimethanil	0.05	1	√	√	√	1.99E-01	86.48	3.02	16.83	20.58
	dinotefuran	2	0.5		√	√		0.00	#DIV/!	0.00	#DIV/!
	dichlorvos	0.2	0.2			√	3.54E-02	64.10	0.21	14.96	20.00
	cyprodinil	0.2	0.5	√	√	√	8.12E-02	95.10	1.98	13.54	22.80
	dimethoate	0.2	0.5		√	√	6.43E-02	42.66	3.68	11.38	15.79
	trichlorfon	0.2	0.2								
	fenitrothion	0.2	0.5					0.00	#DIV/!	25.03	52.55
	isocarbophos	0.05	0.05								
	procymidone	0.2	2			√	1.19E-01	79.60	17.65	5.87	50.06
	penconazole	0.1	0.2		√	√	2.77E-02	116.07	1.02	11.24	25.95
	myclobutanil	1	1	√	√	√	1.77E-01	105.40	1.81	13.97	27.76
	triadimefon	0.05	1	√	√	√	1.29E-01	114.15	2.37	10.29	26.78
	triadimenol	0.2	1		√	√	1.74E-01	124.77	2.56	14.62	19.24
	imazalil	0.5	0.5	√	√	√	1.07E-01	68.91	1.06	18.32	18.35
	clofentezine	0.5	0.5		√	√	3.55E-02	82.35	11.19	5.41	32.51
	diazinon	0.01	0.5	√	√	√	6.11E-02	104.69	3.09	9.77	28.19

	buprofezin	0.5	2.01		√	√	3.82E-01	101.77	2.33	15.60	23.83
	tebuconazole	0.05	2	√	√	√	3.17E-01	108.55	0.17	13.21	22.81
	hexaconazole	0.1	0.5	√	√	√	8.41E-02	110.19	1.59	13.89	23.16
	flusilazole	0.01	0.2	√	√	√	2.19E-02	121.02	0.67	8.76	27.48
	malathion	0.2	0.5	√		√	6.53E-02	113.53	1.94	9.97	29.73
Tomato	bitertanol	0.5	3	√	√	√	4.14E-01	108.31	8.21	10.96	28.50
	propiconazole	0.02	3	√	√	√	5.10E-01	105.20	0.54	13.77	25.21
	hexythiazox	0.05	0.1				5.77E-03	90.80	7.09	4.05	43.33
	profenofos	0.05	10	√	√	√	2.38E+00	98.65	5.20	19.70	22.61
	dimethomorph	0.05	1	√	√	√	2.24E-01	90.03	1.18	19.39	19.07
	difenoconazole	0.02	0.5	√	√	√	6.02E-02	109.24	0.92	9.37	30.95
	spirodiclofen	0.5	0.5		√	√	9.64E-03	80.38	3.41	1.86	47.04
	fenpyroximate	0.1	0.2	√	√	√	1.19E-02	88.46	4.19	4.41	38.02
	parathion-methyl	0.02	0.02				5.02E-04	126.57	10.44	6.73	111.42
Cabbage	demeton (o+s)	0.02	0.02					34.04	11.95	60.59	13.54
	methomyl	0.2	0.2	√	√	√	1.15E-01	18.38	14.27	51.34	16.96
	methamidophos	0.05	0.05			√		3.09	15.90	13.90	76.03
	methidathion	0.05	0.05			√	1.73E-02	52.79	14.56	34.94	1.57
	chlordimeform	0.01	0.01	√	√	√	2.77E-02	222.57	23.69	303.59	12.53
	omethoate	0.02	0.02					5.44	23.37	39.11	12.76
	carbofuran	0.02	0.02				1.25E-02	54.38	13.72	57.95	10.68
	monocrotophos	0.03	0.03	√		√					
	phosfolan-methyl	0.03	0.03				2.58E-02	20.01	13.60	77.97	14.82

	isofenphos-methyl	0.01	0.01	√		√	7.58E-03	84.24	9.29	71.45	8.65
	ethoprophos	0.02	0.02				1.65E-02	77.31	6.52	78.61	6.95
	phosfolan	0.03	0.03				1.96E-02	26.01	10.84	62.06	7.46
	phorate	0.01	0.01				5.75E-03	63.39	12.65	57.60	0.14
Cabbage	cadusafos	0.02	0.02				1.55E-02	106.76	7.76	70.47	13.63
	fenthion	0.05	0.05				1.00E-02	18.15	13.37	20.83	5.02
	parathion	0.01	0.01			√		35.62	49.22	30.20	5.41
	isazofos	0.01	0.01				6.40E-03	103.39	3.56	56.19	19.62
	sulfotep	0.01	0.01				7.39E-03	90.57	7.30	66.84	14.91
	fipronil	0.02	0.02								
	aldicarb	0.03	0.03					18.49	10.02	31.03	2.08
	propamocarb	0.3	10	√		√	4.40E-01	29.84	16.86	38.21	20.28
	fenpropathrin	0.2	1				8.81E-01	117.21	15.37	91.29	4.99
	tebufenozide	0.5	0.5	√	√	√	3.45E-01	102.76	6.88	60.38	20.39
	carbaryl	1	1	√	√	√	6.77E-01	81.19	11.35	63.61	9.10
	teflubenzuron	0.05	0.5	√	√	√	2.73E-01	51.61	22.60	55.87	3.36
	etofenprox	0.6	1			√	8.66E-01	75.47	21.85	86.55	0.19
	permethrin	1	5	√	√	√					
	cypermethrin	1	2								
	cyfluthrin	0.01	0.5								
	pyraclostrobin	0.02	5	√	√	√	3.96E+00	101.44	6.87	76.57	4.70
	carbosulfan	0.05	0.052			√		353.46	35.34	117.47	20.15
	cyhalothrin	0.2	1		√						
	pendimethalin	0.1	0.2				1.82E-01	118.67	9.86	89.93	1.51

	pirimicarb	0.01	1	√	√	√	7.40E-01	61.55	11.18	68.63	11.15
	tau-fluvalinate	0.5	0.5			√	3.49E-01				
	deltamethrin	0.5	0.5				8.11E-01				
	diflubenzuron	1	1		√	√	3.82E+00	80.47	8.85	78.26	5.20
	kresoxim-methyl	0.5	5				1.79E+00	90.07	7.36	69.96	12.63
Cabbage	chlorfluazuron	0.1	2		√	√	3.62E-01	91.95	12.66	89.66	1.18
	tolfenpyrad	0.5	0.5	√	√	√		94.23	12.84	66.77	11.95
	fenvalerate	3	3								
	phoxim	0.05	0.05	√	√		2.72E-02	60.12	19.72	53.03	3.65
	clothianidin	0.5	2		√	√	1.10E+00	53.22	13.80	50.00	13.92
	phosmet	0.05	0.5	√		√	2.21E-01	57.48	14.43	42.66	4.90
	phosalone	1	1	√		√	5.38E-01	57.70	16.31	52.17	4.35
	acephate	0.3	1			√	1.21E-01	5.35	14.62	9.82	32.96
	chlorpyrifos	0.05	0.1				7.24E-02	77.57	20.60	69.90	4.95
	dinotefuran	2	6		√	√	4.11E+00	19.38	8.80	64.00	9.81
	imidacloprid	0.2	0.2			√	1.15E-01	62.18	9.85	53.97	9.03
	dichlorvos	0.2	0.5	√		√	2.74E-01	40.39	7.23	53.34	3.66
	acetamiprid	0.2	1	√	√	√	5.81E-01	60.22	11.12	55.50	6.50
	dimethoate	0.2	1			√	6.05E-01	42.16	12.23	56.43	10.10
	propargite	2	2	√		√	1.56E+00	81.23	18.67	70.00	15.79
	trichlorfon	0.2	2			√	1.27E+00	17.11	14.40	57.23	16.24
	fenitrothion	0.2	0.5				1.90E+00	468.72	10.15	356.92	9.07
	isocarbophos	0.05	0.05			√					
	myclobutanil	1	0.05		√	√	3.25E-02	103.82	9.61	63.53	3.47
	thiamethoxam	0.2	3		√	√	1.68E+00	38.52	14.87	52.48	9.82

	diazinon	0.01	0.05	√		√	3.33E-02	101.02	9.93	63.67	6.53
	tebuconazole	0.05	7	√	√	√	4.97E+00	98.60	7.69	69.13	4.84
	malathion	0.2	8	√		√	5.52E+00	95.60	8.19	67.72	2.79
	difenoconazole	0.02	1	√	√	√	8.27E-01	116.42	4.10	72.40	20.09
	chlorfenapyr	10	2					52.54	4.08	40.67	5.74
Cabbage	parathion-methyl	0.02	0.02				2.19E-02	103.11	13.93	102.91	8.97
Leek	demeton (o+s)	0.02	0.02					13.47	37.99	11.91	44.87
	methomyl	0.2	0.2				4.62E-02	19.06	4.33	17.49	27.94
	methamidophos	0.05	0.05	√		√	2.90E-02	1.63	35.08	2.66	15.96
	methidathion	0.05	0.05	√	√	√		87.84	21.29	35.97	53.60
	chlordimeform	0.01	0.01	√	√	√		0.64	96.48	0.00	#DIV/!
	omethoate	0.02	0.02		√			4.43	24.37	5.24	21.30
	carbofuran	0.02	0.02	√	√	√	3.87E-03	36.12	6.35	13.35	39.77
	monocrotophos	0.03	0.03	√	√	√	6.89E-02	19.28	54.50	193.47	16.81
	phosfolan-methyl	0.03	0.03	√	√	√	9.53E-03	14.07	13.89	26.73	16.63
	isofenphos-methyl	0.01	0.01	√	√	√		47.07	20.02	3.90	27.32
	ethoprophos	0.02	0.02	√	√	√	5.74E-03	89.64	6.14	19.49	42.73
	phosfolan	0.03	0.03	√	√	√	5.01E-03	14.55	7.02	11.76	36.68
	phorate	0.01	0.01				1.43E-03	51.31	7.02	7.65	75.33
	cadusafos	0.02	0.02		√		5.57E-03	97.46	10.34	17.72	50.27
	fenthion	0.05	0.05			√	5.20E-03	37.83	15.64	6.70	50.88
	parathion	0.01	0.01				5.63E-04	36.91	14.28	1.88	173.21

	isazofos	0.01	0.01		√	√	1.53E-03	71.99	6.82	9.29	58.47
	sulfotep	0.01	0.01				1.77E-03	89.75	11.28	10.53	63.12
	fipronil	0.02	0.02								
	Aldicarb	0.03	0.03				3.26E-03	18.30	2.97	6.37	62.21
	Fenpropathrin	0.2	1	√	√	√	2.13E-01	53.23	59.94	12.67	62.56
	Phoxim	0.05	0.05	√		√	1.15E-02	64.17	16.91	14.23	54.14
	Carbaryl	1	1	√	√	√	2.49E-01	64.64	5.15	18.63	30.14
	Teflubenzuron	0.05	0.5		√	√	1.24E-01	35.71	23.72	14.24	64.39
	Etofenprox	0.6	1	√		√	3.41E-01	53.59	4.53	15.23	108.39
	Permethrin	1	1	√	√	√					
	Acephate	0.3	1	√	√	√	1.13E-02	1.05	19.17	0.82	36.53
	Cypermethrin	1	1								
	Cyfluthrin	0.01	0.5								
	Chlorpyrifos	0.05	0.1				2.61E-02	47.19	57.95	13.77	78.35
Leek	Imidacloprid	0.2	1		√	√	1.38E-01	39.55	2.54	8.65	51.78
	Dichlorvos	0.2	0.2	√		√	6.42E-02	40.02	2.02	21.53	43.56
	Carbosulfan	0.05	0.05	√		√	1.63E-03	15.31	10.71	1.92	60.39
	Acetamiprid	0.2	0.02		√	√	2.64E-03	27.30	8.39	8.57	47.20
	Cyhalothrin	0.2	0.5								
	Dimethoate	0.2	0.2		√	√	1.80E-02	20.17	8.20	6.20	39.69
	Pendimethailin	0.1	0.2	√	√	√	4.52E-02	68.55	20.51	11.74	80.51
	tau-Fluvalinate	0.5	0.5								
	Trichorfon	0.2	0.2				6.37E-02	18.87	9.50	20.30	49.51
	Fenitrothion	0.2	0.5				5.43E-01	94.35	8.19	73.31	43.12
	Iscocarbophos	0.05	0.05	√	√	√		30.66	57.16	3.48	173.21

	Procymidone	0.2	0.2		√		2.10E-02	36.72	24.06	28.15	124.92
	Myclobutanil	1	0.06	√	√	√	1.90E-02	90.94	2.68	20.28	49.41
	Boscalid	2	5		√	√	1.69E+00	69.07	1.78	23.42	39.46
	Chlorfluazuron	0.1	1		√		1.59E-01	29.56	60.13	7.70	92.02
	Azoxystrobin	0.1	1	√	√	√	3.39E-01	89.78	1.61	24.10	36.19
Leek	Trifloxystrobin	0.1	0.7	√	√	√	1.87E-01	83.07	2.95	15.67	62.93
	Parathion-methyl	0.02	0.02				1.49E-02	93.68	9.47	44.38	61.63

The letter “a” represents the maximum residue limits (MRLs) of pesticides in agricultural products based on the national standard GB 2763—2021 in China. DL, detection limits. “√” indicate the pesticide is detectable. N.P. indicates non-pretreatment

Table S2. Particle thickness of PDA layer

DA-HCl (mg)	Fe_3O_4@PVP (mg)	Time (h)	magnetism	Thickness (nm)	SD (%)
67	67	24	√	8.04	44.78
200	67	24	√	23.31	25.97
334	67	24	√	33.82	22.12
534	67	24	×	68.42	38.89

SD indicates standard deviation.

Table S3. The preparative composition of different polymers

No.	Template molecule	Templates concentration (mM)	Proportion (Templates: MAA: TRIM)	Qe MIP (mg/g)	Qe NIP (mg/g)	IF
	DDV	0.208		7.61	9.39	0.81
1	FMU	0.208	1:4:15	23.58	20.50	1.15
	CHL	0.208		14.28	12.95	1.10
	DDV	0.208		9.23	6.64	1.39
2	FMU	0.208	1:6:15	19.09	16.66	1.14
	CHL	0.208		14.72	9.93	1.48
	DDV	0.208		15.70	9.21	1.70
3	FMU	0.208	1:6:10	20.19	11.30	1.79
	CHL	0.208		23.57	12.77	1.85

DDV, FMU and CHL represent dichlorvos, flutriafol, and chloromethanes, respectively. MAA and TRIM were methacrylic acid and trimethylolpropane trimethacrylate, respectively.

Table S4. Adsorption isotherm parameters of dichlorovos (DDV), fluometuron (FMU), and chlorotoluron (CHL) onto the MMMIPs and MMNIPs

Templates	Isotherm Models	Equation	Parameter	MMMIPs	MMNIPs
DDV	Langmuir	$\frac{C_e}{Q_e} = \frac{1}{Q_m K_L} + \frac{C_e}{Q_m}$	R^2	0.9757	0.9910
			Q_{max}	21.0526	23.2635
			K_L	0.0025	0.0013
	Freundlich	$\log Q_e = \left(\frac{1}{n}\right) \log C_e + \log K_F$	R^2	0.9708	0.9520
			1/n	0.8373	0.8307
			K_F	0.0843	0.0569
FMU	Langmuir	$\frac{C_e}{Q_e} = \frac{1}{Q_m K_L} + \frac{C_e}{Q_m}$	R^2	0.9963	0.9918
			Q_{max}	42.9185	32.2581
			K_L	0.0310	0.0015
	Freundlich	$\log Q_e = \left(\frac{1}{n}\right) \log C_e + \log K_F$	R^2	0.9680	0.9187
			1/n	0.5631	0.4963
			K_F	0.3635	0.1626
CHL	Langmuir	$\frac{C_e}{Q_e} = \frac{1}{Q_m K_L} + \frac{C_e}{Q_m}$	R^2	0.8920	0.8164
			Q_{max}	19.7239	10.5932
			K_L	0.0129	0.0152
	Freundlich	$\log Q_e = \left(\frac{1}{n}\right) \log C_e + \log K_F$	R^2	0.9321	0.9345
			1/n	0.6650	0.7014
			K_F	0.7539	0.5899

DDV, FMU and CHL represent dichlorvos, flutriafol, and chloromethanes, respectively. (a) Correlation coefficient; (b) Maximum absorption capacity (mg g^{-1}); (c) Langmuir adsorption constant (L mg^{-1}); (d) Freundlich adsorption equilibrium constant (mg g^{-1}); (e) Related to adsorption intensity and surface heterogeneity.

Table S5. Adsorption kinetics parameters of DDV, FMU and CHL onto the MMMIPs and MMNIPs

		Pseudo-first-order			Pseudo-second-order		
		$Q_{e1,\ cal}$ (mg g^{-1})	k_1 (min^{-1})	R_1^2	$Q_{e2,\ cal}$ (mg g^{-1})	k_2 (g mg^{-1} min^{-1})	R_2^2
MMMIPs	DDV	7.7422	0.2088	0.9758	8.0710	0.0388	0.9965
	FMU	15.5795	0.6931	0.9884	15.6986	0.1087	0.9997
	CHL	17.1446	0.4467	0.9662	17.6678	0.0451	0.9971
MMNIPs	DDV	5.6574	0.1786	0.9803	5.9737	0.0418	0.9967
	FMU	8.9665	0.4055	0.9506	9.1659	0.0816	0.9959
	CHL	12.0819	0.2869	0.9669	12.8865	0.0331	0.9982

DDV, FMU and CHL represent dichlorvos, flutriafol, and chloromethanes, respectively. $Q_{e,cal}$ represents theoretically calculated adsorption capacity at equilibrium; k_1 and k_2 belong to the pseudo-first-order rate constant and pseudo-second-order rate constant, respectively. R^2 indicates correlation coefficient.

Table S6. Performance comparison of MMMIPs as pretreatment materials for pesticide detection with other similar studies

Method	Number of analytes	Detection time (min)	Recycling	LOD or SDL ($\mu g\ g^{-1}$)	Application scenarios	Ref.
MMMIPs-LTP-MS	86	15-20	√	The default MRLs of national standard (GB 2763—2021)	4 kinds of vegetables	This study
MMMIPs-LC-TQ-MS	100	30-40	√	0.000043-5.52	4 kinds of vegetables	This study
Biodegradable AgNPs film coupled to UV detection	4	>100	x	0.67-4.12	7 kinds of vegetables	(1)
[MimCH2COOCH3][NTf2]	5	17	x	0.8-2.7	water	(2)
Fluorescence sensor	1	210	x	1.5E-05	2 kinds of vegetables	(3)
TAAEAI-MS	6	<1	x	0.0124-0.0245	strawberry	(4)
FUN-PANI-MIPs	1	>100	√	1.13×10^{-8} M	6 kinds of vegetables	(5)
QuEChERS-GC/MS/MS	208	>30	x	0.01~ 0.05	General foods	(6)

(1) Rovina, K., Vonnie, J. M., Mantihal, S., Joseph, J., & Halid, N. F. A. (2021). Development

of films based on tapioca starch/gold nanoparticles for the detection of organophosphorus pesticides. Journal of Consumer Protection and Food Safety, 16, 143-152.

(2) Lu, D. et al. Rational design of an ionic liquid dispersive liquid–liquid micro-extraction method for the detection of organophosphorus pesticides. Analyst 144, 2166–2172 (2019).

(3) Dong, J., Yang, H., Li, Y., Liu, A., Wei, W., & Liu, S. (2020). Fluorescence sensor for organophosphorus pesticide detection based on the alkaline phosphatase-triggered reaction. Analytica Chimica Acta, 1131, 102-108.

(4) Ma, Z. et al. Tip-assisted ambient electric arc ionization mass spectrometry for rapid detection of trace organophosphorus pesticides in strawberry. Chin. Chem. Lett. 33, 4411–4414 (2022).

(5) Liang, Y. et al. A novel molecularly imprinted polymer composite based on polyaniline nanoparticles as sensitive sensors for parathion detection in the field. Food Control 133, 108638 (2022).

(6) National food safety standard—Determination of 208 pesticides and metabolites residues in foods of plant origin—Gas chromatography-tandem mass spectrometry method (GB 23200.113—2018).
